# Management of pertrochanteric fractures in patients over 90 years: In-hospital mortality rate, complications and associated risk factors

**DOI:** 10.1186/s12891-021-04683-x

**Published:** 2021-09-16

**Authors:** Mohamed Ghanem, Jonas Garthmann, Anja Redecker, Annette Brigitte Ahrberg-Spiegl, Johannes Karl Maria Fakler, Ulrich Josef Albert Spiegl

**Affiliations:** 1 Department of Orthopaedics, Traumatology and Plastic Surgery, University of Leipzig, Liebigstr. 20, 04103 Leipzig, Germany; 2 Department of Physical Therapy and Rehabilitation, University of Leipzig, Leipzig, Germany

**Keywords:** Pertrochanteric fractures, 90-year-old patients, Timing of surgery

## Abstract

**Purpose:**

This study aims primarily to investigate the outcome following surgical management of pertrochanteric fractures of patients over 90 years compared to the outcome of a control group below 90 years under special consideration of the timing of surgery. The second aim was to analyze potential risk factors for early deaths in very old patients. This study allows us to draw conclusions to minimize complications linked to this particular age segment.

**Methods:**

The study group consisted of very old patients aged 90 years and older. Geriatric patients aged between 60 and 89 years of age were part of the control group. Type A1 pertrochanteric fractures were typically treated by dynamic hip crews, type A2 and A3 fractures by femoral nails. Full weight bearing physiotherapy was initiated on the day after surgery to improve mobility and muscle strength.

**Results:**

A total of 71 patients belonged to the study group (mean age: 92.5 years ±2.3 years), whereas 223 patients formed the control group (mean age: 79.9 ± 7.4 years). The mortality rate and the number of detected and documented complications were significantly higher in the study group (*p* = 0.001; *p* = 0.009, respectively). Despite the significantly higher complication rate in the > 90-year-old patients, there was no significant difference in the mean length of in-hospital-stay between the both groups (> 90 yrs.: 12.1d; < 90 yrs.: 13.1 d) and the timing of surgery.

**Conclusion:**

The number of co-morbidities, number of daily-administered medications and the time between admission and surgery have no impact on the outcome. We noticed a longer period between admission and surgery in very old patients who survived. Patients with pertrochanteric fractures should be screened for multimorbidity and cognitive disorders in a standardized manner.

## Introduction

Proximal femoral fracture is one of the most common injuries in old age and is therefore one of the most important causes of morbidity and mortality in the older generation [1–3]. In Germany, around 90,000 proximal femoral fractures are expected annually [4–6]. The age-dependent incidence shows an exponential increase ranging from 60/100,000 in the 60–64-year-old segment up to over 1300/100,000 per year for people over the age of 85 [7]. On an average age of around 80 years, women are being affected in 3 out of 4 cases [8]. Due to the demographic development, several prognoses predict that the number of proximal femoral fractures will quadruplicate by 2050 [2, 3, 8–10]. With constantly increasing life expectancy and the shift in the age pyramid, we see, in addition to the increasing number of cases, the emergence of a new patient collective - that of the “very old”, which confronts treating physicians with new challenges due to the mostly pre-existing multimorbidity. Therefore, we examined the particular age group between octogenarians and centenarians; that age group above 90 years that tends to expand demographically.

A geriatric patient is characterized by geriatric-typical multimorbidity and older age (mostly over 70 years). The so-called geriatric-typical multimorbidity is to be weighted far more heavily than the numerical age [11–13]. Basically, it is represented by immobility, instability, incontinence, and intellectual changes [14]. Yet, patients over 90 years generally present with almost all of the above-mentioned criteria.

In general, fractures can be classified according to the internationally used classification of AO-Foundation, whereby a distinction is made between localization and dislocation [15]. Surgical management of proximal femoral fracture aims at minimizing postoperative pain and complications, immediate mobilization after surgery with full weight bearing of the extremity, rapid return to activities of daily living and prevention from falling. According to German guidelines, patients with pertrochanteric femoral fracture must be operated on within 24 h if the general condition of the patient allows this. This is based on literature reports [16–19] that have shown increased incidence of complications due to postponing surgery for more 24–48 h such as:

- increased morbidity and mortality,
- increased incidence of failure of osteosynthesis,
- increased rates of decubital ulcers and
- increased incidence of venous thrombosis and pulmonary embolism.

However, Zajonz et al. have shown that some geriatric patients with femoral neck fractures might benefit from elongated preoperative treatment protocol [20].

The primary aim of this study is to investigate the outcome following surgical management of pertrochanteric fractures of patients over 90 years compared to the outcome of a control group below 90 years under special consideration of the timing of surgery. Secondly, the aim was to analyze potential risk factors for early deaths in very old patients. This study allows us to draw conclusions to minimize complications linked to this particular age segment.

## Methods

The study was performed at a single level I trauma center between January 2014 and December 2017. All geriatric patients (aged 60 and older) with isolated pertrochanteric femoral fractures after suffering from a low energy trauma were included. Further inclusion parameters were operative treatment of the fracture. All patients, who refused operative treatment and those who died prior surgery and all pathologic fractures due to malignancies were not included. Thereby, the following two study groups were generated: The study group consisted of very old patients aged 90 years and older. Geriatric patients aged between 60 and 89 years of age were part of the control group. The study was approved by the ethics committee of the local university. The vote -number of the audit authority is 166–20-ek. All procedures reported in this article were in accordance with the ethical standards of the institutional and/or national research committee and with the 1964 Helsinki declaration and its later amendments or comparable ethical standards.

Initially, all patients received conventional radiographs of the pelvis and the affected hip joint. In the rare cases of uncertainty of a fracture a computer tomography was performed. The fractures were classified according to the AO [15] and the treatment strategy was defined by the head of the department or the trauma surgeon on duty. Preoperative preparation was started and a rapid operative treatment was desired, ideally within 24 h after admission to the hospital. The following parameters were documented: Fracture classification, treatment strategy, numbers of diagnosis at admission, number of medications at admission, time interval between admission and surgery, and surgery time,. Thereby, the all diagnosis were grouped based on the affected organ and certain diseases as the following: cardiac, vascular, pulmonary, gastroenteric, liver, renal, thyroid, cerebal, spinal pathologies or autoimmune disease, diabetes mellitus, malignancies, obesity, and cachexia.

### Surgical algorithm

Type A1 pertrochanteric fractures were typically treated by dynamic hip crews, type A2 and A3 fractures by proximal femoral nails. Thereby, long femoral nails were typically used in A3 fractures. Concomitant hip osteoarthritis and the individual fracture morphology as well as intraoperative complications had impact on the treatment concept such as performing hip arthroplasty or leading to a change of the strategy of osteosynthesis.

### Postoperative management

All patients received conventional radiographs on the postoperative days 2–4. Full weight bearing physiotherapy was initiated on the day after surgery to improve mobility and muscle strength. Dual X-Ray Absorptiometry (DXA) assessment and sufficient anti-osteoporotic therapy were recommended to all patients.

### Outcome parameters

Patients were followed for the time of the in-hospital stay. The length of the in-hospital stay, all complications, revision surgeries and all deaths were analyzed. Revisions surgeries were defined as any unplanned surgery/ies during the in-patient stay after initial treatment of the fracture. Complications were defined as all documented events during the in-patient stay that were not existent during admission. These included death as the primary outcome parameter and revision surgery as the second outcome parameter as well as venous thrombosis, pulmonary embolism, pneumonia, urinary tract infections, anemia, gastrointestinal bleeding, cardiac events (arterial fibrillation, myocardial infarction); sepsis, decubitus, and electrolyte derailment.

### Statistics

Statistical analyses were performed using standardized SPSS software 25.0 (SPSS®, Inc. Chicago, USA). Statistical analysis was made using descriptive statistics. The mortality rate served as our primary outcome. For secondary outcome parameter we analyzed the rate of revision surgeries, the complication rate as well as the length of the in-hospital-stay. Thereby, all collected parameters were compared between both study groups. For this we took a closer look at the age and gender of the patient, classification and therapy of the proximal femur fracture, number of comorbidities and daily medications as well as time passed between admission and surgery. After an exploratory data analysis, selected parameters were further investigated by Pearson’s test if parameters were distributed normally. Unpaired t-test was employed to compare the study group (patients > 90 years) with the control group (patients 60–89 years old) in normally distributed parameters. A significance level of 0.05 was used.

## Results

A total of 294 geriatric patients were identified with proximal femoral fractures. The study cohort is presented in Table 1.

**Table 1:** Study Cohort

Paremeter	> 90 years; *n* = 71	std	60–89 years; *n* = 223	Std	*p*-value
Age	92.5	2.3	79.7	7.4	< 0.001
Gender [%]	85.9% female		67.7% female		0.003
Classification^a^	1.9 A1 fracture: 21 (30%) A2 fracture: 33 (46%) A3 fracture: 17 (24%)	0.7	1.8 A1 fracture: 83 (37%) A2 fracture: 95 (43%) A3 fracture: (20%)	0.8	0.26
Treatment strategy^b^	1.7 DHS: 30 (42%) PFN: 41 (58%)	0.8	1.6 DHS: 92 (41%) PFN: 124 (56%) BA: 3 (1%) THA: (2%)	0.6	0.90
n/comorbidities	5.2	2.4	4.5	2.8	0.809
n/medications	8.7	2.9	8.6	3.3	0.063
Time: admission to surgery [h]	30.3	43.6	26.0	40.1	0.448
Revisions [%]	9.9		9.0		0.830
Mortality [%]	8.5		0.9		0.001
Complication [%]	41		24		0.005
n/complications	0.6	0.8	0.3	0.7	0.009
In-hospital-stay [days]	12.1	6.7	13.1	9.8	0.408

n/comorbidities: Number of comorbidities; n/medications: number of medications at admission; time: admission to surgery [h]: time between admission to surgery in hours; n/complications: number of complications; std.: standard deviation

^a^ 1: fracture type A1; 2: fracture type A2; 3: fracture type A3

^b^ 1: dynamic hip screw (DHS); 2: proximal femur nail (PFN); 3: bipolar arthroplasty (BA); 4: total hip arthroplasty (THA); 5: others

A total of 71 patients belonged to the study group (mean age: 92.5 years ±2.3 years), whereas 223 patients formed the control group (mean age: 79.9 ± 7.4 years). The female gender dominated in both groups with a significant higher rate in the study group (control group: 67% versus study group: 87%; *p* = 0.003). No differences were seen in the facture classification, the applied treatment strategy, the number of co-morbidities, and the number of daily medications between both groups. In contrast, the mortality rate and the number of detected and documented complications were significantly higher in the study group (*p* = 0.001; *p* = 0.009, respectively). Thereby, there were no significant differences between patients of the study group who died during the hospital stay compared to those who were dismissed alive with respect of age, number of co-morbidities, number of medications, number of complications as well as time between admission and surgery **(**Table 2**)**. A more detailed list of the co-morbidities is illustrated in Table 3. All patients’ parameters of all six patients who died are shown in Table 4.

**Table 2:** Potential risk factors of death in very old patients

Parameters	Death (*n* = 6)	std	No death (*n* = 65)	Std	*P*-value^+*^
Age	92.7	1.8	92.5	2.4	0.664
n/comorbidities	6.0	3.3	5.1	2.4	0.538
n/medications	9.0	4.6	8.7	2.8	0.689
Time: admission to surgery [h]	16.7	13.4	31.3	45.0	0.142
n/complications	0.8	1.0	0.5	0.7	0.448

n/comorbidites: Number of comorbidities; n/medicactions: number of medications at admission; time: admission to surgery [h]: time between admission to surgery in hours; n/complications: number of complications; std.: standard deviation

* Mann-Whitney-U-Test

**Table 3:** Frequency of comorbidities of treated patients in accordance of the affected organ

Parameter	> 90 years; ***n*** = 70	60–89 years; ***n*** = 223
Cardiac disease	31 (44,3%)	91 (40,8%)
Vascular disease	15 (21,4%)	47 (21,1%)
Pulmonary disease	8 (11,4%)	25 (11,2%)
Gastroenteric disease	7 (10%)	23 (10,3%)
Liver disease	3 (4,3%)	6 (2,7%)
Renal disease	26 (37,1%)	35 (15,7%)
Diabetes mellitus	15 (21,4%)	65 (29,1%)
Thyroid dysfunction	11 (15,7%)	51 (22,9%)
Autoimmune disease	5 (7,1%)	4 (1,8%)
Malignancy	7 (10%)	23 (10,3%)
Cerebral disease	6 (8,6%)	26 (11,7%)
Psychic disease	26 (37,1%)	59 (26,5%)
Neurologic disease	11 (15,7%)	37 (16,6%)
Spine	6 (8,6%)	15 (6,7%)
Obesity	1 (1,4%)	11 (4,9%)
Cachexia	1 (1,4%)	1 (0,4%)

**Table 4:** Demographics of the very old patients who died during the inpatient stay

Patient	Age	AO-C	Treatment strategy	Time: admission to surgery	Comorbidities	Complications
1	92	A1	DHS	18 h:09	dementia	ARF, MI
2	90	A1	DHS	12 h:07	dementia; HT; LE.; h/o PE	none
3	92	A2	Nail	8 h:04	DM; HT, COPD	ARF
4	94	A1	Nail	19 h:55	h/o PE; COPD	UTI, pneumonia
5	95	A2	DHS	12 h:03	HT; AF; DM; PAOD	none
6	93	A2	Nail	15 h:12	HT; AF; h/o; malignancy	none

*AO-C* Classification of the fracture in accordance to the AO; *Time: admission to surgery* Time between admission and begin of surgery in hours, *DHS* Dynamic hip screw, *Nail* Proximal femoral nail, *HT* Hypertension, *LE* Lupus erythematodes, *h/o* History of, *PE* Pulmonary embolism, *DM* Diabetes mellitus, *COPD* Chronic obstructive pulmonary disease, *AF* Atrial fibrillation, *PAOD* Peripheral artery occlusive disease, *ARF* Acute renal failure, *MI* Myocardial infarction, *UTI* Urinary tact infection

**Fig. 1:**
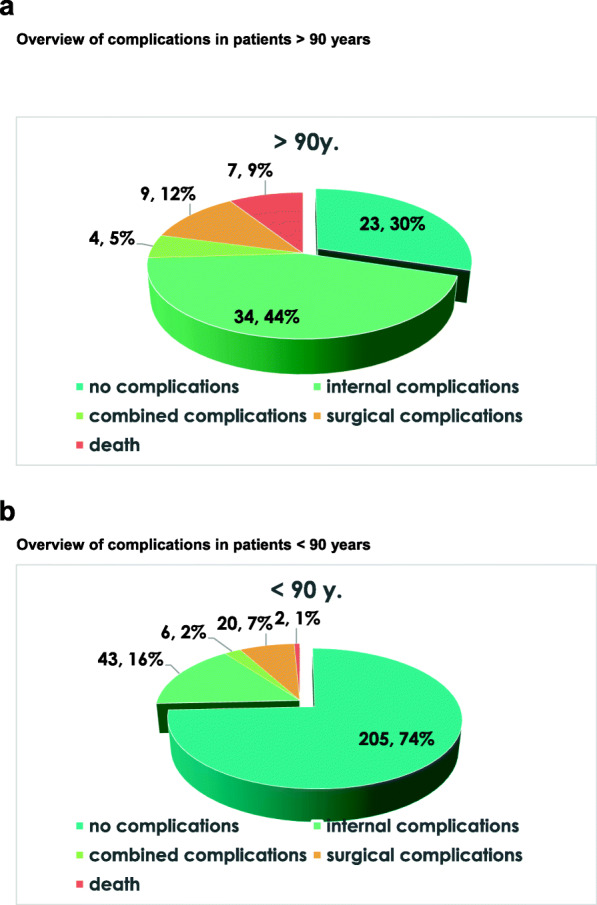
Overview of complications in patients > 90 years

In the control group 170 of 223 (76.2%) showed no complications following surgical treatment whereas the proportion of uncomplicated courses in study group was only 42 of 71 (59.2%). Most of the complications documented in both groups were general complications, particularly of urinary tract infections, anemia, pneumonia or acute deterioration of renal function. In contrast, surgical complications, particularly postoperative wound healing disorders and secondary failure of osteosynthesis was less frequently seen. Despite the significantly higher complication rate in the > 90-year-old patients, there was no significant difference in the mean length of in-hospital-stay between the both groups (> 90 yrs.: 12.1d; < 90 yrs.: 13.1 d) and the timing of surgery. Thereby, the average time between admission and surgery was 16.7 h in the very old patients who died and 31.3 h in those who survived the hospital stay, without being statistical significant (*p* = 0.14). All further potential risk factors that were analyzed (age, number of comorbidities, number of medications on admission, number of complications) were statistically not different between survivors and deaths of the study group. An overview of all complications of the study group is shown in Fig. 1. There was no significant difference with respect of the revision rate between the two study groups with 7 of 71 (9.9%) in the study group compared to 20 of 223 (9.0%) in the control group.

## Discussion

In this retrospective study, we compared the outcome following surgical management of pertrochanteric fractures of patients over 90 years compared to the outcome of a control group aging 60 to 89. Particular attention was given to the overall complications, the impact of the number of co-morbidities, the number of daily medications as well as the time interval between hospital admission and surgical intervention on the outcome.

We observed a significantly higher incidence of mortality, general complications as well as surgery-related complications in patients > 90 years. The number of co-morbidities, number of medications, number of complications as well as time between admission and surgery did not have a significant impact of the outcome. This shows that an age above 90 years predisposes to higher mortality rate as well as to a higher rate of perioperative complication.

Though there are several reports in literature on proximal femoral fracture of the elderly, to the best of our knowledge, we did not encounter a comparative study that addresses this issue concerning the particular age group above 90 years.

Costa et al. evaluated data from over 16,000 patients after head replacement surgery for femoral neck fractures [21]. 6.7% of the patients died in the hospital.

Based on studies of a collective of over 22,000 patients [22, 23] and a further retrospective study of around 38,000 patients [24], a hospital mortality ranged from 4.5 to 6.6%.

In our study, we only examined the rate and type of in-hospital complications. Several reports dealt with short term overall complication after discharge from hospital. Yet, the particular age segment above 90 years has not been analyzed specifically. Based on 93 re-examined patients after a femoral fracture near the hip joint, Simanski et al. reported a mortality after 12 months of 33% with a mean age of 74.7 years [25]. Raunest et al. also calculated the 1-year mortality after a proximal femur fracture [26]. With an average age of 78.7 years, they reported 27.3% who died within 12 months. A 1-year mortality of 11.7% was given by Röder et al. [2]. Smektala et al. examined the prognosis of a femoral neck fracture [23]. They found a mortality after 12 months of 24.2%. Lögters et al. published a 1-year mortality rate of 35.3% with a high average age of 86 years [10]. In their study, Holvik et al. stated a 1-year mortality rate of 23.5%, with a high mean age of 85 years [27]. Smektala et al. confirmed that patients with a higher ASA classification have a significantly poorer survival [23]. Holvik et al. also examined the difference in the ASA classification between the survivors and the deceased after 1 year [27]. With 46.2% compared to 69.2%, significantly fewer surviving study patients had the ASA III or IV classification. Daugaard et al. were able to show that an increasing ASA classification means a risk increase for the outcome “death during inpatient stay” of 2.3 per level [24]. Davis et al. confirmed the influence of the ASA level on mortality, as did Uzoigwe et al. [3, 28].

In light of our analysis and the above-mentioned literature reports as well as further reports [16–19, 29, 30], we can state that it is highly recommendable to surgically treat patients over 90 years with proximal femoral fracture in hospitals that are equipped with maximum infrastructure for dealing with such cases. Though many literature reports notably stress on the necessity to operate within less than 24 h [16–19, 29, 30], we could not observe any benefits in those patients treated within the first 24 h of admission. Interestingly, the time period between admission and surgery was clinically significantly shorter in very old patients who died and those who survived without being statistically different. This might suggest that a thorough preoperative conditioning can lower the incidence of postoperative mortality in patients over 90. This was also observed by Zajonz et al. [20]. Therefore, patients over 90 years with proximal femoral fracture should be optimally prepared for surgery even though this might prevent operative stabilization within 24 h after admission. Additionally, patients will benefit from a hospital setup that is able to provide sufficient surgical experience, medical, well-educated anesthesiologic and nursing staff for very old patients and an adequate infrastructure as well as intensive care capacity. The intersection of geriatric traumatology with other specialist disciplines, especially acute geriatrics, will be of enormously increasing importance in the future.

Schiavone et al. [31] investigated the use of tranexamic acid in the management of peritrochanteric femoral fracture. They concluded that the use of tranexamic acid was statistically significant in reducing postoperative blood loss. Yet, the mortality in the study population of patients over 75 was linked more to the chronic inflammatory state and comorbidities rather than to the use of tranexamic acid.

Studies have shown that periprosthetic or postoperative infections can shorten life expectancy [32, 33]. Falzarano et al. suggest the use of seriated controls of C-reactive protein, erythrocyte sedemantion rate and procalcitonin for patients undergoing total hip arthroplasty in the first 4 weeks after surgery to detect and manage infection as early as possible. The regular investigation of these parameters should be considered in management of proximal femoral fractures to monitor patients concerning infection as possible complication that can have impact on life expectancy, especially in the elderly.

Concerning stability of the proximal femur following surgery, studies did not show a direct relation between the degree of stability and life expectancy [34, 35]. Lanzetti et al. demonstrated that in intertrochanteric 31-A1 and 31-A2 stable fractures, the absence of distal locking screw does not compromise bone healing and prevents several clinical complications. Yet, Maiettini et al. concluded that increasing the use of blinded assessment of outcomes and improved reporting of reliability of subjective end points will improve the quality of interferences derived from clinical studies.

The currently available data do not allow for drawing definite conclusion concerning the impact of revision surgery on life expectancy. Yet, some studies retrospective studies have shown a higher tendency of poor functional outcomes and complications that led to death following revision surgery in elderly patients [36–38].

The limitation of this study lies in its retrospective design and the short follow-up period that is limited to in-hospital stay. Though we considered the number of daily medications administered and have shown no impact on the outcome, we did not analyze the type of medications due to the heterogeneity of medications administered. However, to the best of our knowledge, very few studies have dealt with proximal femoral fractures in patients over 90 years [39, 40]. In contrast to these studies, we included a control group. Yet, the control group comprised of patients aged 60 to 90, which bares considerable heterogeneity and thus must be viewed as a further limitation. Thus, further studies with comparison with similar age and gender can reveal more precise data.

## Conclusion

Surgical management of pertrochanteric fractures in patients over 90 years seems to be associated with higher incidence of postoperative complications. According to our study, the number of co-morbidities, number of daily-administered medications and the time between admission and surgery might have no impact on the outcome. Contrary to previous studies, we noticed a longer period between admission and surgery in very old patients who survived. Therefore, further studies are needed to analyze the outcome of surgical management of pertrochanteric fractures in the 10th decade of life, particularly with regards to timing of surgical intervention and the extent of preoperative conditioning. Further, patients with pertrochanteric fractures should be screened for multimorbidity and cognitive disorders in a standardized manner.

## Data Availability

The datasets used and/or analyzed during the current study are available from the corresponding author on reasonable request.

## Abbreviations

AO: Arbeitsgemeinschaft für Osteosynthesefragen
ASA: American Society of Anesthesiologists
ICD: International Statistical Classification of Diseases and Related Health Problems
h: Hour
